# Blocking Brain Development: How PCBs Disrupt Thyroid Hormone

**Published:** 2005-07

**Authors:** Valerie J. Brown

Polychlorinated biphenyls (PCBs) have long been known to alter growth and development in animals and humans, and are suspected of interfering with the action of thyroid hormone (TH) in humans. Much less is known about which congeners of this large chemical family may have such action and how they interfere with TH. Now, in an *in vitro* study using human brain stem cells, a team of German and Californian researchers shows how a specific PCB congener, PCB-118, can interrupt normal TH function and cause the premature differentiation of one type of brain cell **[*EHP* 113:871–876]**.

Many animal studies, primarily in rats, suggest that PCBs can profoundly affect fetal brain development, which itself is highly dependent on the availability and amount of TH, principally from the mother. PCBs are known to lower circulating blood levels of TH by increasing TH metabolism and binding to TH transport proteins; the exact mechanisms and effects of TH disruption by PCBs are unclear, however. Some epidemiologic evidence suggests a link between PCB exposure during fetal development and subsequent cognitive problems in children, such as lowered overall IQ, attention and motor deficits, and impaired impulse control. The suspicion that these problems may be related to PCBs’ effects on the timing and type of brain cell differentiation led to the current study.

Stem cells known as normal human neural progenitor (NHNP) cells develop into three types of brain cells: neurons, which receive and transmit electrical signals via axons and synapses; astrocytes, which manage neurons’ surrounding environment; and oligodendrocytes, which produce myelin, the fatty sheath that insulates axons. TH is known to control the timing of oligodendrocyte differentiation.

The research team exposed NHNP cells to two PCB congeners—PCB-118 and PCB-126—and observed the effects on cell differentiation. They found that cells exposed to PCB-118 prematurely turned into oligodendrocytes. This finding suggests strongly that PCBs may facilitate the binding of coactivator proteins to cellular TH receptors; these proteins then mimic the action of TH. Paradoxically, a surplus of oligodendrocytes early in brain development may lead to a dearth later, because if there are proportionally fewer neurons, many oligodendrocytes cannot wrap an axon or reproduce. In that case, they undergo apoptosis, or programmed cell death. The end result would likely be a drop in the total number of brain cells.

The current study is consistent with an earlier rat study published in the April 2004 issue of *EHP*, which found that PCBs interfered directly with fetal TH receptor signaling, as opposed to reducing circulating maternal TH levels. Like many other studies, that study used Aroclor 1254, a commercial mixture of several PCB congeners, and did not determine the effects of each congener. The current study compared two PCB congeners that differ somewhat in their toxic equivalence (TEQ). The results showed that the lower-TEQ congener (PCB-118) acted via the TH pathway while the higher-TEQ congener (PCB-126) did not. The apparent toxicity of a PCB congener with a low TEQ also suggests that the TEQ system may not be a reliable measure of all types of toxicity.

